# Association of different types of milk with depression and anxiety: a prospective cohort study and Mendelian randomization analysis

**DOI:** 10.3389/fnut.2024.1435435

**Published:** 2024-12-05

**Authors:** Chunying Wu, Yusheng Liu, Yigui Lai, Qiang Wang, Siqi Wu, Huijie Fan, Yanyan Liu, Xiaoshan Zhao, Xuefeng Jiang

**Affiliations:** ^1^Department of Central Laboratory, People's Hospital of Yangjiang, Yangjiang, China; ^2^School of Traditional Chinese Medicine, Southern Medical University, Guangzhou, China

**Keywords:** milk, depression, anxiety, UK biobank, Mendelian randomization

## Abstract

**Background:**

The relationship between different types of milk and depression and anxiety remains unclear, with limited evidence from prospective cohort studies. This study aims to evaluate this relationship using data from the UK Biobank cohort and to explore its potential causality through Mendelian randomization (MR) analysis.

**Methods:**

Cox proportional hazards models were used to assess the association between different milk types and the risk of depression and anxiety among 357,568 UK Biobank participants free of these conditions at baseline. To further explore causality, a 2-sample MR analysis was conducted using data from the FinnGen study.

**Results:**

During a median follow-up period of 13.5 years (interquartile range, 12.6–14.2 years), among 357,568 participants (mean [SD] age, 56.83 [8.06] years, 171,246 male individuals [47.9%]), a total of 13,065 and 13,339 participants were diagnosed with depression and anxiety, respectively. In the fully adjusted model (adjusted for sociodemographics characteristics, lifestyle behaviors and health indicators), full cream milk was related to a lower risk of anxiety (HR = 0.84, 95% CI: 0.75–0.94). Semi-skimmed milk had a lower risk of depression (HR = 0.88, 95% CI: 0.80–0.96) and anxiety (HR = 0.90, 95% CI: 0.82–0.98). No significant relationships were found between skimmed milk and depression/anxiety. Other types were related to an increased risk of depression (HR = 1.14, 95% CI: 1.02–1.28). After Bonferroni correction, the 2-sample MR analysis revealed a potential protective causal relationship between semi-skimmed milk and depression (OR = 0.83, 95% CI: 0.73–0.95, *p* = 0.006) and anxiety (OR = 0.71, 95% CI: 0.59–0.85, *p* < 0.001).

**Conclusion:**

These findings indicate that semi-skimmed milk consumption may be linked to a lower risk of depression and anxiety, potentially highlighting its role in dietary strategies to promote mental health.

## Introduction

Mental disorders are increasingly recognized as major causes of disease burden, among which depression and anxiety disorders are the most common. Globally, it is estimated that 3.8% of the population is afflicted with depression (1), and 4% experience an anxiety disorder (2). The prevalence of depression among young people (10–24 years) has also increased sharply over the past decade (3). Depression and anxiety disorders not only increase healthcare costs, but also raise the risk of chronic physical conditions such as cardiovascular disease (CVD), diabetes mellitus, chronic lung disease, and chronic pain (4). Moreover, depression and anxiety disorders are strongly associated with an increased risk of suicide (5). Currently, treatments for mental health mainly center on psychological therapy and medication. Nevertheless, these treatments only prevent less than half of the disease burden, and many people are unable to access their benefits.

Evidence from observational and intervention studies in nutritional psychiatry indicates that diet plays a significant role on mental health (6–8). Milk is a widely consumed dairy product worldwide, encompassing full cream, semi-skimmed, skimmed, and other varieties. A study on dietary patterns based on the UK Biobank reported that individuals in the highest milk intake group exhibited an increased prevalence of mental health symptoms (9), yet the relationship between different types of milk and mental health was not further investigated. A previous study of 1,159 Japanese individuals aged 19–83 years found that compared with those who did not consume semi-skimmed dairy, the odds ratios (OR) and 95% confidence intervals (CI) for moderate frequency (1–3 times/week) was 0.96 (95% CI: 0.71–1.30), and 0.51 (95% CI: 0.35–0.77) for high frequency (≥4 times/week) (10). However, another cross-sectional study conducted on 7,387 Iranian adults (20–69 years) reported that the consumption of high-fat milk was associated with a reduced risk of depression (OR = 0.73, 95% CI: 0.59–0.91) after adjusting for potential confounding factors, while the intake of semi-skimmed milk was related to a decreased likelihood of anxiety symptoms (OR = 0.82, 95% CI: 0.68–0.99) (11). Thus, the association between different types of milk and depression and anxiety remains unclear, and previous observational studies mainly utilized cross-sectional designs, lacking prospective studies.

This study aims to evaluate the associations of different types of milk with depression and anxiety in a prospective cohort study. Additionally, we conducted a 2-sample MR analysis, a statistical approach that utilizes genetic variants as instrumental variables to examine the causal effect of exposure on an outcome (12), in order to explore the potential causal relationship between different types of milk and depression and anxiety.

## Methods

### Study design and population

The UK Biobank is a long-term prospective cohort study that includes biological and medical data collected on 502,402 adult participants (37–73 years) from the UK between 2006 and 2010, with multiple follow-ups. With the consent of the participants, information such as physical measurements, genotype data, biological samples (such as blood and urine), imaging data, and detailed lifestyle information is obtained periodically and then linked to their health-related records. The UK Biobank has received approval from the North West Multi-centre Research Ethics Committee (13, 14).

**Figure 1:**
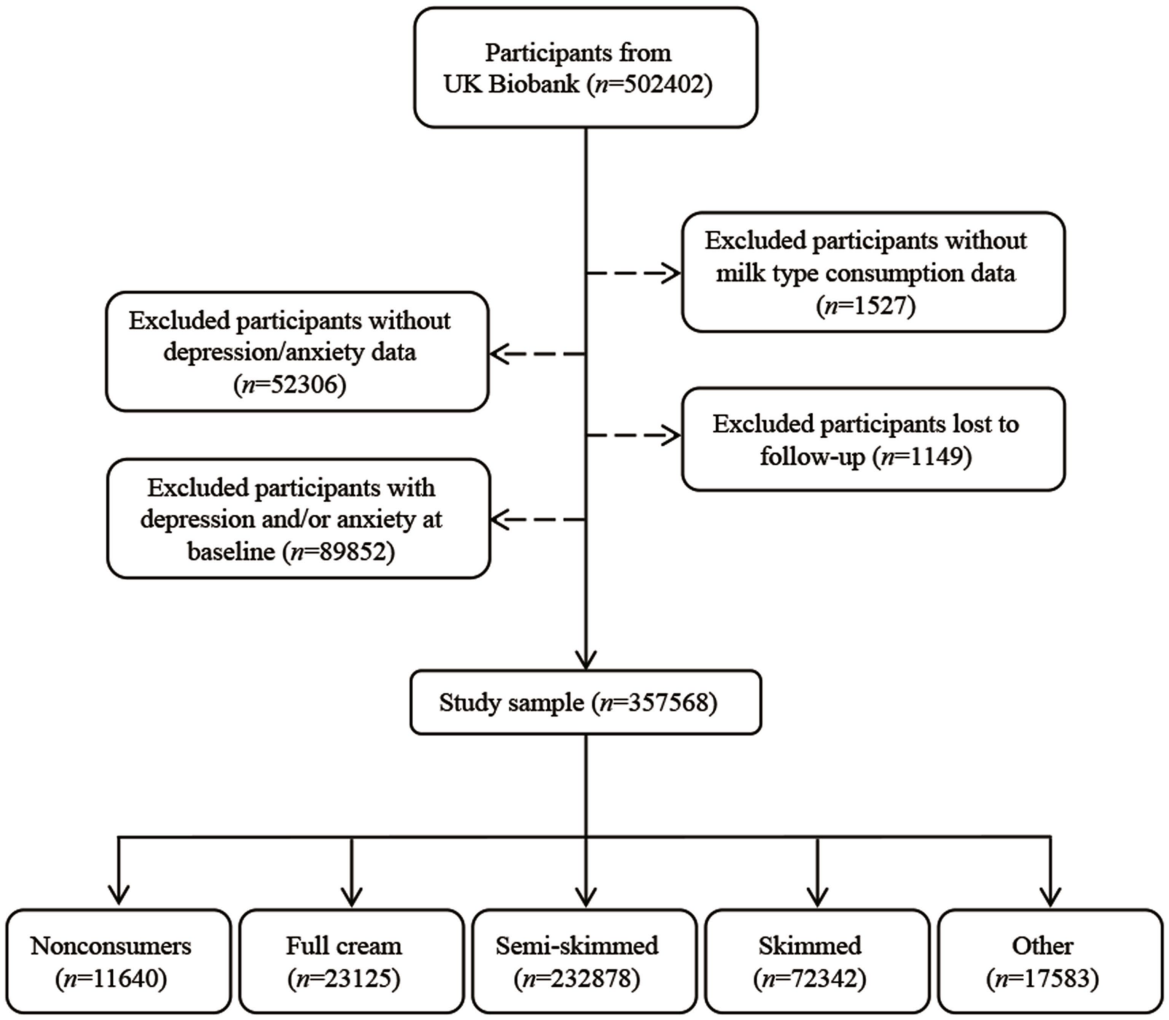
Study sample diagram.

In this study, participants with information on milk types consumption at baseline were eligible for inclusion. We excluded those with no information on milk types consumption at baseline (*n* = 1,527), those who did not take part in the Patient Health Questionnaire (PHQ)-4 questionnaire at baseline (*n* = 52,306), those who were lost to follow-up (*n* = 1,149), and those with depression or anxiety at baseline (*n* = 89,852). Finally, 357,568 participants were included in this study (Figure 1).

### Assessment of exposure

Information on milk type consumption was obtained using a touchscreen questionnaire at baseline. Participants were asked, “What type of milk do you mainly use?” and given a list of options, including full cream, semi-skimmed, skimmed, soya (categorized as “other types of milk” in this study), other types of milk, never/rarely consume milk, do not know, and prefer not to answer. Participants who answered “do not know” or “prefer not to answer” were considered missing data. We excluded 1,527 participants who had missing data regarding milk type consumption.

### Assessment of outcomes

At baseline, depression and anxiety were evaluated using the PHQ-4 questionnaire (15) and the date of the initial reporting of depression or anxiety. For the follow-up, depression and anxiety were assessed via the PHQ-9 questionnaire (16) or the Generalized Anxiety Disorder (GAD)-7 questionnaire (17), along with the date of the first reported. Individuals were considered to have depression or anxiety if either of these records were positive. For outcomes classification, the International Classification of Diseases-10th revision (ICD-10) coding system was employed. In this analysis, we measured depression (F32-F33) and anxiety (F40-F41). For participants who did not experience depression or anxiety at baseline, the follow-up time was estimated based on the date of the first reported or the date of censoring (September 9, 2022), whichever occurred first.

The PHQ-4 questionnaire is a simplified form of the PHQ-9 and GAD-7 questionnaires, consisting of 4 main items. Participants completed a touchscreen questionnaire in which they rated their thoughts and feelings over the past 2 weeks on a scale of 0 (not at all) to 3 (nearly every day). A score of ≥3 on items 1 and 2 indicated depression, and a score of ≥3 on items 3 and 4 indicated anxiety. During the follow-up period, the participants’ mental health was measured between 2016 and 2017 using the PHQ-9 and GAD-7 questionnaires. The PHQ-9 questionnaire comprises 9 items, while the GAD-7 questionnaire has 7 items, both are scored on a scale from 0 (not at all) to 3 (nearly every day) using the same four-point Likert scale. A total score of ≥10 is considered positive for depression or anxiety, respectively.

### Assessment of covariates

We used the baseline touchscreen questionnaire and medical records to assess the following potential confounders: sociodemographics characteristics (age, sex, income, ethnicity, education level), lifestyle behaviors (smoking status, physical activity, intake of vegetables, fruits, coffee, and alcohol), and health indicators (body mass index (BMI), hypertension, diabetes, CVD, cancer). Physical activity indicates whether a person met the 2017 UK Physical activity guidelines of 150 min of moderate activity per week or 75 min of vigorous activity. The BMI value was constructed from height and weight measured at baseline. The history of hypertension was collected from the date of first reported and baseline blood pressure measurements. Diabetes and CVD were determined based on the date of the first reported. Cancer data was obtained from self-reported medical conditions and doctor-diagnosed data at baseline and the date of cancer diagnosis in the cancer register.

### 2-sample MR analysis

We performed a 2-sample MR analysis to investigate the potential causal effects of consuming semi-skimmed milk (UK Biobank field id 1,418) on depression and anxiety risk. Exposure data for the analysis of semi-skimmed milk consumption were obtained from genome-wide association study (GWAS) summary statistics in the UK Biobank,^1^ which included 456,422 individuals of European ancestry (18). The GWAS summary statistics for depression and anxiety were sourced from the FinnGen study (R10, *n* = 412,181 individuals).^2^ The FinnGen study is a large-scale genomics initiative that has analyzed over 500,000 Finnish biobank samples and correlated genetic variation with health data to understand disease mechanisms and predispositions (19). For FinnGen R10, GWAS summary data of 47,696 depression cases and 359,290 controls were included, and GWAS summary data of 27,664 anxiety cases and 368,054 controls were also included.

To obtain valid independent exposure instrumental variables, we used single-nucleotide polymorphism (SNP) with genome-wide significance (*p* < 5e−6) and then performed linkage disequilibrium analyses (*r*^2^ = 0.001, clumping distance = 10,000 kb). One incompatible SNP (rs10944537) was removed after harmonization. Consequently, 27 valid and independent genome-wide significant SNPs were included in the MR analysis. The random-effects inverse variance-weighted (IVW) method (20) was primarily used for the main effect to estimate the potential causal effects of consuming semi-skimmed milk on the risk of depression and anxiety. Weighted median and MR-Egger regression were used as complementary analyses. To assess potential pleiotropy bias in genetic instrumental variables, we utilized two statistical methods: the MR-Egger intercept test (21), which was used to assess the presence of horizontal pleiotropy effect; the MR-Pleiotropy Residual Sum and Outlier (MR-PRESSO) test (22) was used to identify and remove outliers. Additionally, we conducted Cochran’s Q statistics (23) and leave-one-out analyses to assess the robustness of significant results. All statistics analyses were performed using the R (v.4.3.2) packages: TwoSampleMR and MR-PRESSO. The Bonferroni correction was employed to address the issue of multiple testing. Therefore a threshold of *p* < 0.025 (0.05/2) was regarded as strong evidence of associations.

### Statistical analysis

Regarding the missing covariates, we used chained-equation multiple imputation to multiply impute the missing data on the covariates five times (Supplementary Table S1). All variables, including outcomes, were included in the multiple imputation model. Baseline characteristics were presented as mean ± standard deviation (SD) for continuous variables and as numbers (%) for categorical variables. We used Cox proportional risk regression to estimate hazard ratios (HR) and 95% CI for the prospective associations of milk type consumption with depression and anxiety disorders. Four models were constructed: Model 1 was adjusted for age (continuous, years) and sex (female or male). Model 2 was further adjusted for ethnicity (White or other), income (<18,000, 18,000–30,999, 31,000–51,999, 52,000–100,000, >100,000), and education level (degree or no degree). Model 3 was additionally adjusted for smoking status (never, previous, or current), physical activity (yes or no), vegetable intake (continuous, tablespoons/d), fruit intake (continuous, pieces/d), coffee intake (continuous, cups/d), and alcohol intake frequency (<1, 1–2, 3–4, >4, times/week). Model 4 was further adjusted for BMI (<25.0, 25.0 to <30.0, ≥30.0 kg/m^2^), hypertension (yes or no), diabetes (yes or no), CVD (yes or no), and cancer (yes or no).

To assess the robustness of the findings, we conducted several additional analyses. First, subgroup analyses were used to investigate the correlation between milk types and the risk of depression and anxiety. Second, three sensitivity analyses were performed: we excluded participants who experienced an outcome event within the first 2 years of follow-up to avoid reversal of causality; we excluded participants with any missing covariates (Supplementary Table S1); we excluded participants with a history of CVD or cancer at or before baseline. Finally, associations between milk types and the risk of depression and anxiety were assessed using the Fine-Gray competing risk model (24). Statistical significance was set at *p* < 0.05. All analyses were performed using R software, version 4.3.2.

## Results

### Prospective cohort study

#### Baseline characteristics

A total of 357,568 participants were included in this study, having a mean age of 56.8 ± 8.1 years. Among them, 171,246 (47.9%) were male, and 186,322 (52.1%) were female. Additionally, 11,640 (3.3%) reported being non-milk consumers, and 345,928 (96.7%) were milk consumers. Semi-skimmed milk (232,878, 65.1%) was the most commonly consumed type, followed by skimmed milk (72,342, 20.2%), full cream milk (23,125, 6.5%), and other type of milk (17,583, 4.9%). Non-consumers of milk were more likely to be drinking coffee. Full cream milk consumers were more likely to be male, had less income, and be current smokers. Semi-skimmed milk consumers were less likely to be eating vegetables and fruit and more likely to be drinking alcohol 3 to 4 times per week. Skimmed milk consumers were more likely to be older, white, previous smokers, obese, and had hypertension, diabetes, and CVD (Table 1).

**Table 1:** Baseline characteristics of UK Biobank participants in the prospective cohort analysis.

Characteristics	Overall		Non-consumers		Milk consumers	
			Full cream	Semi-skimmed	Skimmed	Other
Participants, *n*	357,568 (100.0)	11,640 (3.3)	23,125 (6.5)	232,878 (65.1)	72,342 (20.2)	17,583 (4.9)
Age, years	56.8 (8.1)	56.6 (8.0)	56.2 (8.4)	56.7 (8.1)	57.6 (7.9)	56.5 (8.0)
Sex, male	171,246 (47.9)	5,210 (44.8)	14,567 (63.0)	117,345 (50.4)	28,286 (39.1)	5,838 (33.2)
**Income, £**						
<18,000	59,548 (19.2)	1,918 (18.9)	5,427 (27.2)	37,430 (18.5)	11,582 (18.7)	3,191 (21.4)
18,000–30,999	77,800 (25.1)	2,320 (22.9)	5,352 (26.8)	50,307 (24.9)	15,948 (25.7)	3,873 (25.9)
31,000–51,999	83,477 (27.0)	2,643 (26.1)	4,877 (24.4)	55,303 (27.3)	16,657 (26.8)	3,997 (26.8)
52,000–100,000	69,109 (22.3)	2,437 (24.1)	3,420 (17.1)	46,319 (22.9)	13,878 (22.4)	3,055 (20.5)
>100,000	19,511 (6.3)	809 (8.0)	903 (4.5)	13,009 (6.4)	3,974 (6.4)	816 (5.5)
Ethnicity, white	342,260 (96.0)	11,002 (95.1)	21,005 (91.3)	224,017 (96.4)	70,221 (97.3)	16,015 (91.5)
Education, degree	123,181 (35.0)	4,738 (41.2)	7,975 (35.1)	79,582 (34.7)	24,043 (33.8)	6,843 (39.5)
**Smoking status**						
Never	198,658 (55.7)	5,753 (49.6)	12,013 (52.1)	130,444 (56.2)	40,294 (55.9)	10,154 (57.9)
Previous	125,474 (35.2)	4,389 (37.8)	6,822 (29.6)	80,552 (34.7)	27,381 (38.0)	6,330 (36.1)
Current	32,451 (9.1)	1,459 (12.6)	4,219 (18.3)	21,271 (9.2)	4,462 (6.2)	1,040 (5.9)
Physical activity	166,326 (55.9)	5,416 (55.3)	10,782 (56.5)	107,289 (55.3)	33,796 (56.2)	9,043 (60.9)
Vegetables (tablespoons/d)	4.9 (3.3)	5.6 (4.4)	4.5 (3.6)	4.8 (3.1)	5.3 (3.3)	5.8 (3.7)
Fruit (pieces/d)	2.3 (1.6)	2.4 (1.8)	1.8 (1.6)	2.2 (1.5)	2.5 (1.6)	2.6 (1.7)
Coffee (cups/d)	2.0 (2.0)	2.2 (2.5)	2.1 (2.3)	2.0 (2.0)	2.1 (2.0)	1.4 (1.6)
**Alcohol intake frequency**						
<1	97,157 (27.2)	3,159 (27.2)	7,272 (31.5)	59,583 (25.6)	20,655 (28.6)	6,488 (36.9)
1–2	94,159 (26.3)	2,433 (20.9)	5,298 (22.9)	62,322 (26.8)	19,572 (27.1)	4,534 (25.8)
3–4	88,842 (24.9)	2,692 (23.1)	4,700 (20.3)	59,750 (25.7)	17,897 (24.7)	3,803 (21.6)
>4	77,292 (21.6)	3,350 (28.8)	5,839 (25.3)	51,154 (22.0)	14,198 (19.6)	2,751 (15.7)
**BMI (kg/m**^**2**^**)**						
<25.0	121,366 (34.1)	4,082 (35.2)	9,382 (40.8)	78,031 (33.6)	22,000 (30.5)	7,871 (45.0)
25.0 to <30.0	154,628 (43.4)	4,657 (40.2)	9,378 (40.8)	101,768 (43.9)	32,067 (44.5)	6,758 (38.6)
≥30.0	80,165 (22.5)	2,843 (24.5)	4,226 (18.4)	52,195 (22.5)	18,036 (25.0)	2,865 (16.4)
Hypertension	95,211 (26.6)	3,246 (27.9)	5,160 (22.3)	61,918 (26.6)	20,928 (28.9)	3,959 (22.5)
Diabetes	17,181 (4.8)	554 (4.8)	913 (3.9)	11,369 (4.9)	3,707 (5.1)	638 (3.6)
CVD	39,304 (11.0)	1,292 (11.1)	2,222 (9.6)	25,417 (10.9)	8,658 (12.0)	1,715 (9.8)
Cancer	44,659 (12.5)	1,501 (12.9)	2,649 (11.5)	28,620 (12.3)	9,369 (13.0)	2,520 (14.3)

BMI, body mass index; CVD, cardiovascular disease.

#### Types of milk with depression and anxiety

During a median follow-up of 13.5 years (interquartile range, 12.6–14.2 years), 13,065 (3.65%) and 13,339 (3.73%) participants were diagnosed with depression and anxiety for the first time, respectively. We first investigated whether there was an association between milk consumption and depression and anxiety. The results showed that compared to non-consumers, milk consumers had a lower risk of depression and anxiety (Supplementary Table S2).

We conducted a further study to investigate the association between different types of milk and the risk of depression and anxiety. In the basic model, adjusted for age and sex (model 1), consumption of semi-skimmed milk was associated with a lower risk of depression (HR = 0.84, 95% CI: 0.76–0.92) compared to non-consumers. Moreover, full cream milk (HR = 0.87, 95% CI: 0.78–0.98), semi-skimmed milk (HR = 0.88, 95% CI: 0.80–0.96), and skimmed milk (HR = 0.91, 95% CI: 0.82–1.00) were associated with a lower risk of anxiety. In the fully adjusted model (model 4), full cream milk was associated with a lower risk of anxiety (HR = 0.84, 95% CI: 0.75–0.94). Semi-skimmed milk had a lower risk of depression (HR = 0.88, 95% CI: 0.80–0.96) and anxiety (HR = 0.90, 95% CI: 0.82–0.98). No relationships were found between skimmed milk and depression or anxiety. Other types were related to an increased risk of depression (HR = 1.14, 95% CI: 1.02–1.28) (Table 2).

**Table 2:** Association of types of milk with depression and anxiety in the UK Biobank cohort.

Outcomes	Non-consumers	Milk consumers			
		Full cream	Semi-skimmed	Skimmed	Other
**Depression, HR (95% CI)**					
Event, *n* (%)	486 (4.2)	852 (3.7)	8,057 (3.5)	2,863 (4.0)	807 (4.6)
Model 1	1.00 (Reference)	0.94 (0.84–1.05)	0.84 (0.76–0.92)	0.93 (0.85–1.03)	1.06 (0.94–1.18)
Model 2	1.00 (Reference)	0.86 (0.77–0.96)	0.83 (0.76–0.91)	0.93 (0.85–1.03)	1.04 (0.93–1.17)
Model 3	1.00 (Reference)	0.86 (0.77–0.96)	0.87 (0.79–0.95)	0.98 (0.89–1.08)	1.09 (0.97–1.22)
Model 4	1.00 (Reference)	0.91 (0.81–1.02)	0.88 (0.80–0.96)	0.97 (0.88–1.07)	1.14 (1.02–1.28)
**Anxiety, HR (95% CI)**					
Events, *n* (%)	488 (4.2)	766 (3.3)	8,406 (3.6)	2,855 (4.0)	824 (4.7)
Model 1	1.00 (Reference)	0.87 (0.78–0.98)	0.88 (0.80–0.96)	0.91 (0.82–1.00)	1.05 (0.94–1.18)
Model 2	1.00 (Reference)	0.82 (0.73–0.92)	0.86 (0.79–0.95)	0.90 (0.82–0.99)	1.04 (0.93–1.16)
Model 3	1.00 (Reference)	0.82 (0.73–0.92)	0.89 (0.81–0.98)	0.93 (0.84–1.02)	1.06 (0.94–1.18)
Model 4	1.00 (Reference)	0.84 (0.75–0.94)	0.90 (0.82–0.98)	0.93 (0.84–1.02)	1.07 (0.96–1.20)

Model 1 (basic model): Adjusted for age, sex. Model 2: Adjusted for Model 1 plus ethnicity, income, and education. Model 3: Adjusted for Model 2 plus smoking status, physical activity, vegetable, fruit, coffee, and alcohol intake frequency. Model 4 (fully adjusted model): Adjusted for Model 3 plus BMI, hypertension, diabetes, CVD, and cancer. HR, hazard ratios; BMI, body mass index; CVD, Cardiovascular disease.

#### Subgroup and sensitivity analyses

**Figure 2:**
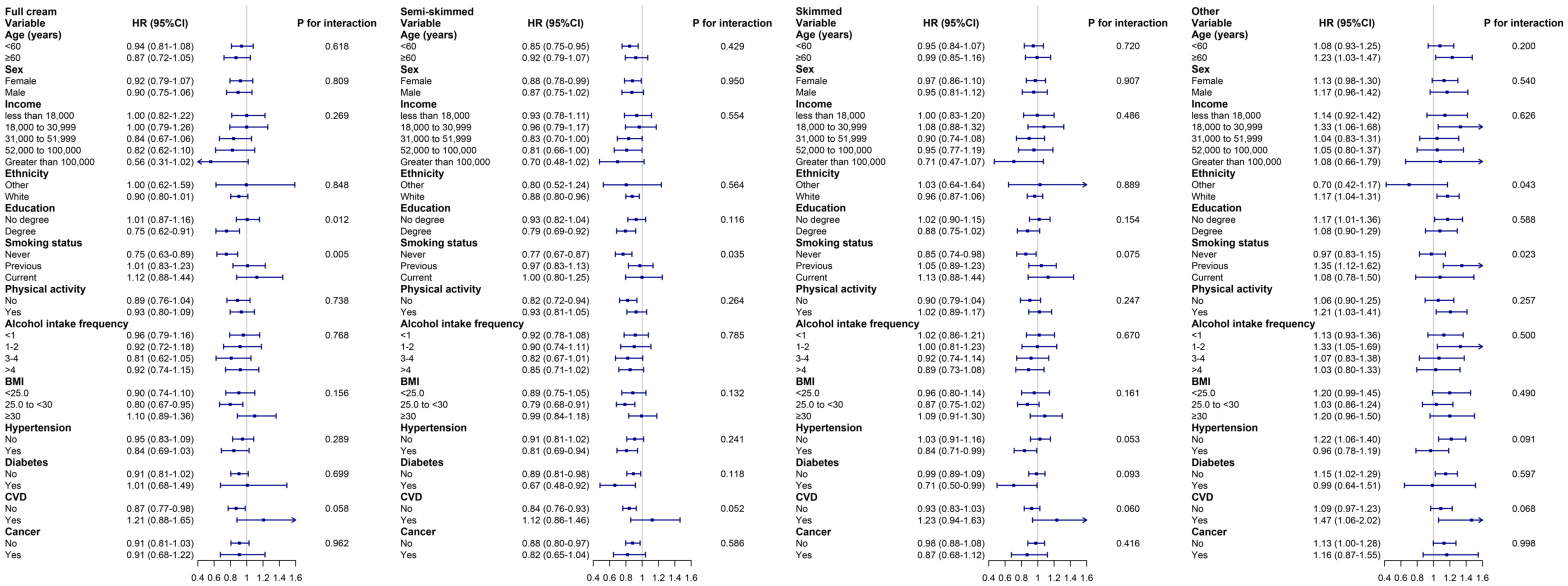
Associations of types of milk consumption with depression by subgroups. Adjusted for age, sex, ethnicity, income, education, smoking status, physical activity, vegetable, fruit, coffee, alcohol intake frequency, BMI, hypertension, diabetes, CVD, and cancer. HR, hazard ratios; BMI, body mass index; CVD, Cardiovascular disease.

Subgroup analyses were performed based on age (<60 or ≥60), sex (female or male), ethnicity (White or other), income (<18,000, 18,000–30,999, 31,000–51,999, 52,000–100,000, >100,000), education level (degree or no degree), smoking status (never, previous, or current), BMI (<25.0, 25.0 to <30.0, ≥30.0 kg/m^2^), physical activity (yes or no), alcohol intake (<1, 1–2, 3–4, >4, times/week), hypertension (yes or no), diabetes (yes or no), CVD (yes or no) and cancer (yes or no). The results indicated that the associations between milk consumption and depression and anxiety were not significantly different (*P* for interaction >0.05), except for specific outcomes when grouped by education, smoking status, and diabetes (Figure 2; Supplementary Figure S1). The findings remained relatively unchanged following sensitivity analyses, including: (1) the removal of participants who experienced an outcome event within the initial 2 years of follow-up (Table 3); (2) the exclusion of individuals with missing covariates (Supplementary Table S3); (3) the exclusion of participants with CVD or cancer at or before baseline (Supplementary Table S4). Furthermore, competing risk models yielded results comparable to the principal multivariate Cox regression analysis (Supplementary Table S5).

**Table 3:** Association of types of milk with depression and anxiety after excluding participants who experienced an outcome event within the initial 2 years of follow-up.

Outcomes	Non-consumers	Milk consumers			
		Full cream	Semi-skimmed	Skimmed	Other
**Depression, HR (95% CI)**					
Event, *n* (%)	469 (4.0)	836 (3.6)	7,776 (3.3)	2,780 (3.9)	779 (4.4)
Model 1	1.00 (Reference)	0.95 (0.85–1.07)	0.84 (0.76–0.92)	0.94 (0.85–1.04)	1.06 (0.94–1.18)
Model 4	1.00 (Reference)	0.93 (0.83–1.04)	0.88 (0.80–0.96)	0.97 (0.88–1.07)	1.14 (1.02–1.28)
**Anxiety, HR (95% CI)**					
Events, *n* (%)	473 (4.1)	736 (3.2)	8,115 (3.5)	2,767 (3.8)	794 (4.5)
Model 1	1.00 (Reference)	0.87 (0.77–0.97)	0.87 (0.80–0.96)	0.90 (0.82–1.00)	1.05 (0.93–1.17)
Model 4	1.00 (Reference)	0.83 (0.74–0.94)	0.89 (0.81–0.98)	0.93 (0.84–1.02)	1.07 (0.95–1.20)

Model 1 (basic model): Adjusted for age, sex. Model 4 (fully adjusted model): Adjusted for age, sex, ethnicity, income, education, smoking status, physical activity, vegetable, fruit, coffee, alcohol intake frequency, BMI, hypertension, diabetes, CVD, and cancer. HR, hazard ratios; BMI, body mass index; CVD, Cardiovascular disease.

#### 2-sample MR analysis

In our prospective study, we found that the consumption of semi-skimmed milk was associated with a lower risk of both depression and anxiety. Therefore, we conducted a 2-sample MR analysis to examine whether there was a possible causal relationship between semi-skimmed milk and depression and anxiety.

(1)   Semi-skimmed milk and depression

**Figure 3:**
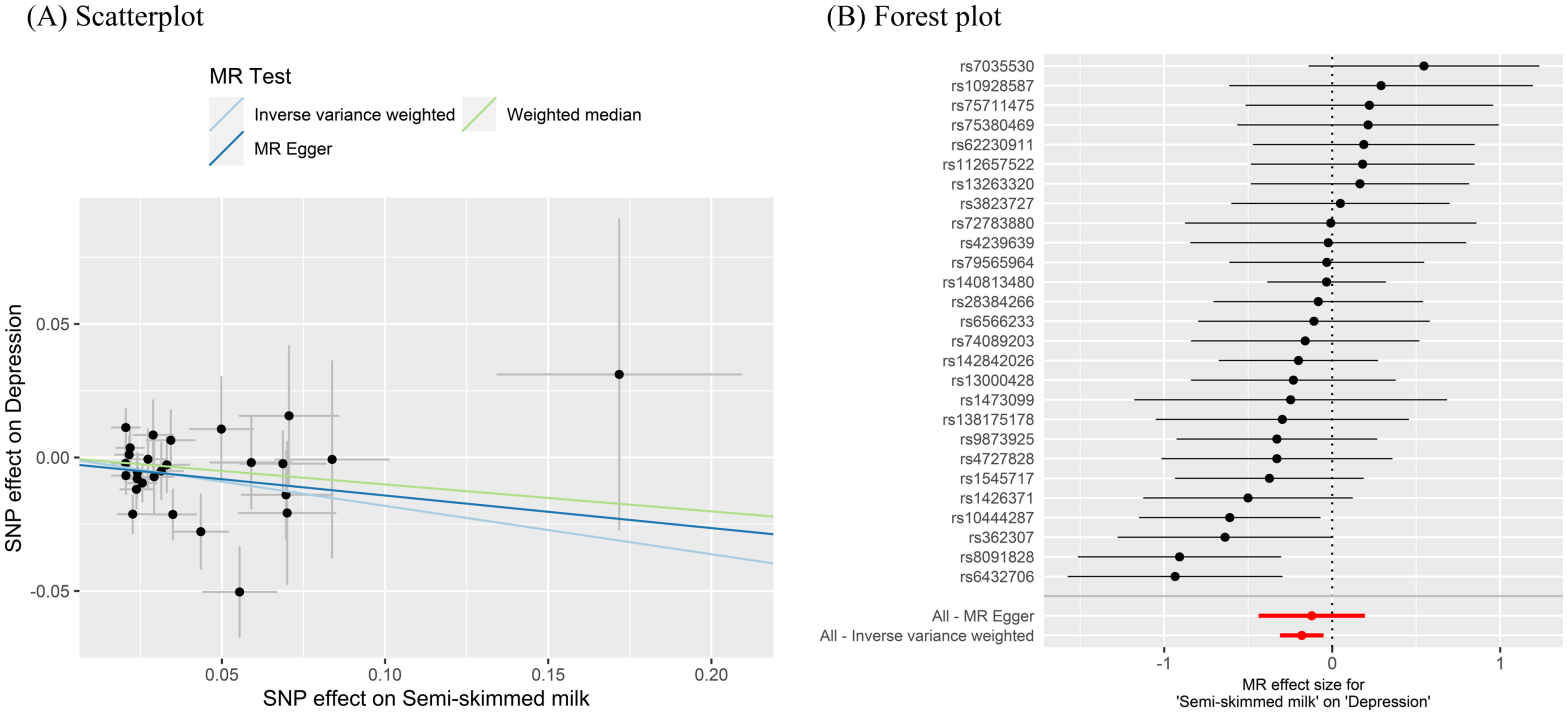
MR Plots for semi-skimmed milk on depression. **(A)** Scatterplot of potential SNP effects on semi-skimmed milk vs. depression, with the slope of each line corresponding to the estimated MR effect per method. **(B)** Forest plot of individual and combined SNP MR-estimated effects sizes. MR, Mendelian randomization; SNP, single-nucleotide polymorphism.

**Figure 4:**
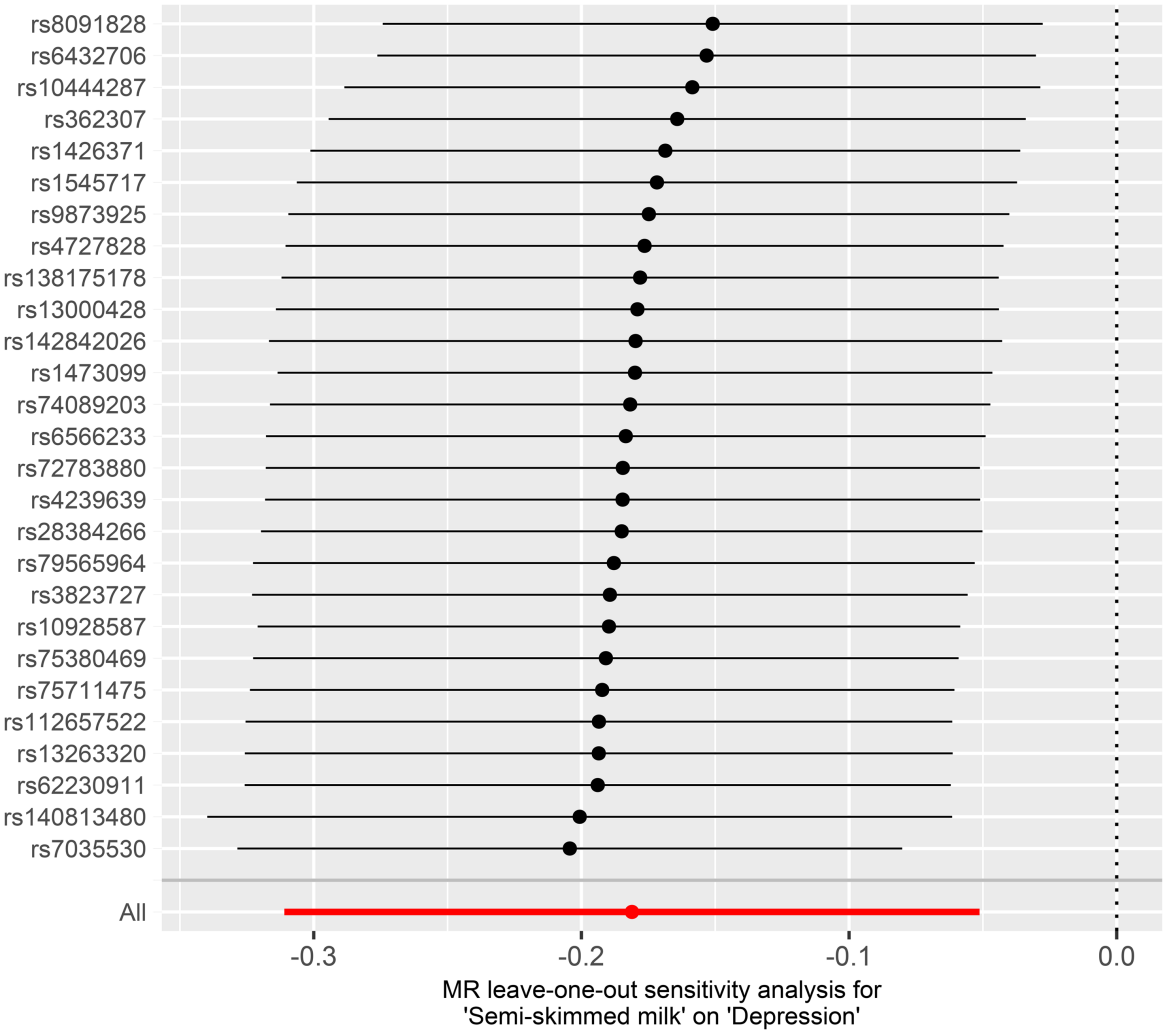
Leave-one-out MR estimates for the effect of semi-skimmed milk on depression. MR, Mendelian randomization; SNP, single-nucleotide polymorphism.

In the 2-sample MR, after Bonferroni correction, the IVW estimate provided evidence of a protective causal relationship between semi-skimmed milk and depression (OR = 0.83, 95% CI: 0.73–0.95, *p* = 0.006) (Table 4; Figure 3). The weighted median and MR-Egger analysis provided similar causal estimates. MR-Egger intercept analyses did not find any horizontal pleiotropy (Intercept = −0.002, *p* = 0.69), and the MR-PRESSO test found no pleiotropic outlier SNPs. Cochran’s Q statistics revealed no significant heterogeneity (*p* = 0.27). In addition, the leave-one-out analysis indicated that no single SNP drove the causal signal (Figure 4).

(2)   Semi-skimmed milk and anxiety

**Table 4:** MR results for the relationship between semi-skimmed milk and depression or anxiety.

Exposure	Outcome	SNP (*n*)	Method	OR (95% CI)	*p*
Semi-skimmed milk	Depression	27	IVW	0.83 (0.73–0.95)	0.006
		27	Weighted median	0.90 (0.75–1.08)	0.277
		27	MR-Egger	0.89 (0.64–1.21)	0.457
Semi-skimmed milk	Anxiety	26	IVW	0.71 (0.59–0.85)	0.000
		26	Weighted median	0.71 (0.56–0.90)	0.005
		26	MR-Egger	0.83 (0.52–1.33)	0.452

MR results used the random-effects inverse-variance weighted model. MR, Mendelian randomization; SNP, single-nucleotide polymorphism; OR, odds ratios; IVW, inverse variance-weighted.

MR-PRESSO test identified a pleiotropic outlier SNP (rs140813480). After the outlier was removed and the Bonferroni correction was applied, we found that semi-skimmed milk was also causally associated with anxiety (IVW, OR = 0.71, 95% CI: 0.59–0.85, *p* = 0.000), the weighted median and MR-Egger analysis showed similar results (Table 4; Supplementary Figure S2). MR-Egger intercept analyses did not identify any horizontal pleiotropy (Intercept = −0.005, *p* = 0.47), and the MR-PRESSO test did not identify any pleiotropic outlier SNPs. Cochran’s Q statistics showed no significant heterogeneity (*p* = 0.24). Furthermore, we performed a leave-one-out analysis, which revealed that no single SNP drove these results (Supplementary Figure S3).

## Discussion

As an essential part of the human diet, dairy products are consumed by over 6 billion people worldwide (25). A study on milk consumption among Americans reported that full cream milk consumption was positively associated with depressive symptoms, while skimmed milk was negatively associated with depressive symptoms, and there was no association between semi-skimmed milk and depressive symptoms among 21,924 adults aged ≥18 years from National Health and Nutrition Examination Survey (26). Meghan et al. revealed that consumption of full cream dairy products (OR = 0.64, 95% CI: 0.41–0.998) was correlated with decreased odds of having elevated depressive symptoms, but there were no significant associations between semi-skimmed dairy and depressive symptoms among 1,600 Finnish adults (mean ages 63 ± 6 years) (27). Another observational study of 311 young undergraduate students (mean age 20.5 years) from Portugal found that consumption of full cream milk, semi-skimmed milk, and skimmed milk was not associated with anxiety (28). After adjusting for sociodemographic characteristics, lifestyle behaviors, and health indicators, our prospective study found that full cream milk only had a connection with a lower risk of anxiety, while semi-skimmed milk was associated with a lower risk of depression and anxiety. No significant relationships were found between skimmed milk and depression or anxiety. The results of our study do not entirely align with or contradict previous research. This may be due to several factors, including the predominantly European population in our study, the age range of our participants (36–73 years), the relatively small sample size in the previous study, and the fact that our study was a prospective cohort study. In addition, subgroup analyses indicated that the associations between milk consumption and depression and anxiety did not differ significantly, except for specific outcomes when categorizing by education, smoking status, and diabetes.

MR studies use genetic variation associated with modifiable exposures to assess their potential causal relationship with outcomes. The intention is to reduce potential bias due to confounding and reverse causation. Due to its ability to provide more reliable evidence of causality than traditional observational studies, MR has become a widely used method in epidemiology (29). The effects of full cream, semi-skimmed, and skimmed milk on human health have been a subject of debated. In our study, we found that consumption of semi-skimmed milk was associated with a reduced risk of depression and anxiety through a prospective cohort study. To strengthen causal inference, we conducted a 2-sample MR analysis with genetic instruments selected from large-scale GWAS to estimate the causal relationship between semi-skimmed milk and depression and anxiety. The MR results suggested that semi-skimmed milk had a protective effect on both depression and anxiety. The results of Cochran’s Q statistic and leave-one-out analysis were similar to the results of the main MR analysis, further supporting the causality of semi-skimmed milk on depression and anxiety. Recently, Shuai Yuan et al. conducted MR studies on the health effects of milk consumption in the European population. The MR results revealed that genetically predicted milk consumption was negatively associated with hypercholesterolemia. A systematic review of MR studies identified additional negative associations between milk consumption and Alzheimer’s disease as well as blood lipid levels, but positive associations with Parkinson’s disease, metabolic syndrome, overweight, obesity, and BMI (30).

Milk is a rich source of nutrients such as lactose, lipids, protein, and minerals, which are essential for maintaining human health (31). The serotonin (5-hydroxytryptamine, 5-HT) system is an important target for correcting numerous central nervous system diseases, including depression, anxiety, and schizophrenia (32). Milk casein can significantly reduce chronic mild stress-induced changes in serum corticosterone and serotonin levels and prevented stress-induced anxiety-like behavior (33). Milk is a good source of calcium that is easily assimilated by the body. Earlier studies have shown that calcium activates tryptophan hydroxylase in the biosynthetic pathway, leading to serotonin synthesis, and the calcium/calmodulin-dependent system may increase dopamine synthesis in the brain (34, 35). A prospective study from China revealed that supplemental calcium intake can decrease the risk of depressive symptoms in older adults (36). The acidity, stability, and casein content of milk are influenced by the varying fat levels present (37). Much of the fat in milk is saturated fat. Among the three milk types, full cream milk contains higher levels of saturated fatty acids, while semi-skimmed milk offers a moderately reduced amount (38, 39). Excessive intake of saturated fats is associated with elevated levels of circulating saturated long-chain fatty acids, such as palmitate, which are positively correlated with the severity of depression (40). The potential mechanisms may involve suppression of dopaminergic function and signaling, enhanced neuroinflammation, and altered leptin levels within the central nervous system (41–43). Additionally, semi-skimmed milk contains significant amounts of unsaturated fats acids (39). Studies suggest that monounsaturated fats help maintain the integrity of the brain’s dopamine system (41), whereas supplementation with omega-3 polyunsaturated fatty acids has shown benefits in depressed patients, including promoting neurogenesis and neural repair, preventing neuroinflammation and neurodegeneration, and improving mood and cognitive functions (44–46). However, the content of unsaturated fatty acids in skim milk is lower than that in semi-skimmed milk (39). Consequently, the fatty acid profile of semi-skimmed milk might provide greater cerebral protection compared to full cream milk and skimmed milk, thereby potentially reducing the risk of both depression and anxiety. Although the protective association of semi-skimmed milk with lower risks of depression and anxiety needs to be further explored. For this purpose, ingredient analyses such as mass spectrum need to be conducted, and well-designed biological experiments should be performed based on the study results.

## Strengths and limitations

This study has several notable strengths. Firstly, it included 357,568 adult participants, constituting a large sample size. Secondly, to our knowledge, this is the first study to combine a prospective cohort design with the MR analysis to explore the associations between various milk types and the risks of depression and anxiety. Thirdly, we developed four models to control for potential confounders by adjusting for socio-demographic, lifestyle, and health-related factors. Furthermore, we validated the results of the prospective cohort study using a 2-sample MR analysis method.

However, our study has several limitations. Initially, baseline milk consumption was self-reported. As health status and individual dietary habits may change over time, this could result in measurement errors in assessing milk consumption. Moreover, although our models incorporated several dietary variables such as intake of vegetables, fruits, coffee, and alcohol, we did not include total energy intake, which is available from the UK Biobank and is known to potentially confound dietary associations. This omission might lead to residual confounding, affecting the interpretation of our findings. We acknowledge this limitation and suggest that future research should consider including total energy intake to more accurately evaluate the relationships studied. Additionally, emotional disorders are prevalent among 10–19 year olds worldwide, with 14% of children and adolescents experiencing mental health conditions (47). Since the participants of the UK Biobank were middle-aged or older, we were unable to obtain more results on the relationship of milk types with depression and anxiety in the youth population. Lastly, the participants in the UK Biobank were predominantly of European descent. Therefore, our findings may not apply to all populations.

## Conclusion

In conclusion, the results of our prospective cohort study and two-sample MR analysis indicate the possibility of an inverse association between the consumption of semi-skimmed milk and the risks of depression and anxiety. These findings suggest that semi-skimmed milk may have a protective effect against these mental health conditions, presenting new prospects for dietary interventions. However, considering that these conclusions are based on self-reported dietary data and are influenced by the extensive duration of the follow-up, the findings must be interpreted cautiously. To robustly establish these associations, further research is needed to validate these results and explore the impact of amount of milk consumed on mental health.

## Data Availability

Publicly available datasets were analyzed in this study. This data can be found at: https://www.ukbiobank.ac.uk/.

## Footnotes

^1^https://yanglab.westlake.edu.cn/data/ukb_fastgwa/imp/

^2^https://r10.finngen.fi/
