# Supplementary material for: Association of different types of milk with depression and anxiety: a prospective cohort study and Mendelian randomization analysis
Source: Front Nutr. 2024 Dec 5;11:1435435. doi: 10.3389/fnut.2024.1435435 (PMC11656347; doi:10.3389/fnut.2024.1435435)
Supplement: Supplementary file 1 [file Table_1.DOCX]

Association of Different Types of Milk with Depression and Anxiety: A Prospective Cohort Study and Mendelian Randomization Analysis

Chunying Wu, Yusheng Liu, et al.

**Supplementary Tables**

This file includes:

[Supplementary Table 1. The numbers (percentages) of participants with missing covariates 2](#_Toc18777)

[Supplementary Table 2. Association of milk consumption with depression and anxiety in the UK Biobank cohort 3](#_Toc31453)

[Supplementary Table 3. Association of types of milk with depression and anxiety after excluding participants with missing covariates 4](#_Toc4059)

[Supplementary Table 4. Association of types of milk with depression and anxiety after excluding participants with CVD or Cancer at or before baseline 5](#_Toc9719)

[Supplementary Table 5. Association of types of milk with depression and anxiety: results from competing risk regression models 6](#_Toc12112)

# Supplementary Table 1. The numbers (percentages) of participants with missing covariates

| **Covariates** | **n** | **%** |
| --- | --- | --- |
| Income | 48,123 | 13.46% |
| Ethnicity | 982 | 0.27% |
| Education | 5,387 | 1.51% |
| Smoking status | 985 | 0.28% |
| Physical activity | 59,807 | 16.73% |
| Vegetable | 1,276 | 0.36% |
| Fruit | 749 | 0.21% |
| Coffee | 344 | 0.10% |
| Alcohol intake frequency | 118 | 0.03% |
| BMI | 1,409 | 0.39% |

BMI, body mass index.

# Supplementary Table 2. Association of milk consumption with depression and anxiety in the UK Biobank cohort

| **Outcomes** | **Non-consumers** | **Milk consumers** |
| --- | --- | --- |
| **Depression, HR (95% CI)** |  |  |
| Event, *n* (%) | 486 (4.2) | 12,579 (3.6) |
| Model 1 | 1.00 (Reference) | 0.88 (0.80-0.96) |
| Model 2 | 1.00 (Reference) | 0.86 (0.79-0.95) |
| Model 3 | 1.00 (Reference) | 0.90 (0.82-0.99) |
| Model 4 | 1.00 (Reference) | 0.91 (0.83-1.00) |
| **Anxiety, HR (95% CI)** |  |  |
| Events, *n* (%) | 488 (4.2) | 12,851 (3.7) |
| Model 1 | 1.00 (Reference) | 0.89 (0.82-0.98) |
| Model 2 | 1.00 (Reference) | 0.88 (0.80-0.96) |
| Model 3 | 1.00 (Reference) | 0.90 (0.83-0.99) |
| Model 4 | 1.00 (Reference) | 0.91 (0.83-1.00) |

Model 1 (basic model): Adjusted for age, sex. Model 2: Adjusted for Model 1 plus ethnicity, income, and education. Model 3: Adjusted for Model 2 plus smoking status, physical activity, vegetable, fruit, coffee, and alcohol intake frequency. Model 4 (fully adjusted model): Adjusted for for Model 3 plus BMI, hypertension, diabetes, CVD, and cancer. HR, hazard ratios; BMI, body mass index; CVD, Cardiovascular disease.

# Supplementary Table 3. Association of types of milk with depression and anxiety after excluding participants with missing covariates

| **Outcomes** | **Non-consumers** | **Milk consumers** | | | |
| --- | --- | --- | --- | --- | --- |
|  |  | **Full cream** | **Semi-skimmed** | **Skimmed** | **Other** |
| **Depression, HR (95% CI)** |  |  |  |  |  |
| Event, *n* (%) | 360 (4.2) | 586 (3.5) | 5,768 (3.4) | 2,055 (3.9) | 610 (4.8) |
| Model 1 | 1.00 (Reference) | 0.89 (0.78-1.02) | 0.81 (0.73-0.90) | 0.92 (0.83-1.03) | 1.10 (0.96-1.25) |
| Model 4 | 1.00 (Reference) | 0.86 (0.76-0.99) | 0.85 (0.76-0.94) | 0.95 (0.85-1.07) | 1.17 (1.03-1.33) |
| **Anxiety, HR (95% CI)** |  |  |  |  |  |
| Events, *n* (%) | 351 (4.1) | 525 (3.1) | 5,987 (3.5) | 1,974 (3.7) | 596 (4.7) |
| Model 1 | 1.00 (Reference) | 0.85 (0.75-0.98) | 0.88 (0.79-0.98) | 0.89 (0.80-1.00) | 1.08 (0.94-1.23) |
| Model 4 | 1.00 (Reference) | 0.82 (0.72-0.94) | 0.90 (0.80-1.00) | 0.91 (0.81-1.02) | 1.09 (0.95-1.24) |

Model 1 (basic model): Adjusted for age, sex. Model 4 (fully adjusted model): Adjusted for age, sex, ethnicity, income, education, smoking status, physical.activity, vegetable, fruit, coffee, alcohol intake frequency, BMI, hypertension, diabetes, CVD, and cancer. HR, hazard ratios; BMI, body mass index; CVD, Cardiovascular disease.

# **Supplementary Table 4.** Association of types of milk with depression and anxiety after excluding participants with CVD or Cancer at or before baseline

| **Outcomes** | **Non-consumers** | **Milk consumers** | | | |
| --- | --- | --- | --- | --- | --- |
|  |  | **Full cream** | **Semi-skimmed** | **Skimmed** | **Other** |
| **Depression, HR (95% CI)** |  |  |  |  |  |
| Event, *n* (%) | 366 (4.0) | 634 (3.4) | 5,948 (3.3) | 2,097 (3.8) | 597 (4.4) |
| Model 1 | 1.00 (Reference) | 0.90 (0.79-1.02) | 0.81 (0.73-0.91) | 0.92 (0.82-1.03) | 1.04 (0.91-1.18) |
| Model 4 | 1.00 (Reference) | 0.87 (0.76-0.99) | 0.85 (0.77-0.95) | 0.95 (0.85-1.07) | 1.11 (0.97-1.27) |
| **Anxiety, HR (95% CI)** |  |  |  |  |  |
| Events, *n* (%) | 364 (4.1) | 578 (3.2) | 6,310 (3.5) | 2,145 (3.8) | 606 (4.5) |
| Model 1 | 1.00 (Reference) | 0.85 (0.75-0.97) | 0.88 (0.79-0.97) | 0.92 (0.83-1.03) | 1.04 (0.91-1.18) |
| Model 4 | 1.00 (Reference) | 0.82 (0.72-0.94) | 0.90 (0.81-1.00) | 0.95 (0.85-1.06) | 1.06 (0.93-1.20) |

Model 1 (basic model): Adjusted for age, sex. Model 4 (fully adjusted model): Adjusted for age, sex, ethnicity, income, education, smoking status, physical.activity, vegetable, fruit, coffee, alcohol intake frequency, BMI, hypertension, diabetes, CVD, and cancer. HR, hazard ratios; BMI, body mass index; CVD, Cardiovascular disease.

# **Supplementary Table 5.** Association of types of milk with depression and anxiety: results from competing risk regression models

| **Outcomes** | **Non-consumers** | **Milk consumers** | | | |
| --- | --- | --- | --- | --- | --- |
|  |  | **Full cream** | **Semi-skimmed** | **Skimmed** | **Other** |
| **Depression, HR (95% CI)** |  |  |  |  |  |
| Event, *n* (%) | 486 (4.2) | 852 (3.9) | 8,057 (3.5) | 2,863 (4.0) | 807 (4.6) |
| Model 1 | 1.00 (Reference) | 0.93 (0.83-1.04) | 0.84 (0.77-0.92) | 0.94 (0.86-1.04) | 1.06 (0.95-1.19) |
| Model 4 | 1.00 (Reference) | 0.90 (0.81-1.01) | 0.88 (0.81-0.97) | 0.98 (0.89-1.08) | 1.15 (1.02-1.29) |
| **Anxiety, HR (95% CI)** |  |  |  |  |  |
| Events, *n* (%) | 488 (4.2) | 766 (3.3) | 8,406 (3.6) | 2,855 (4.0) | 824 (4.7) |
| Model 1 | 1.00 (Reference) | 0.87 (0.77-0.97) | 0.88 (0.81-0.97) | 0.91 (0.83-1.01) | 1.06 (0.95-1.19) |
| Model 4 | 1.00 (Reference) | 0.84 (0.75-0.94) | 0.90 (0.83-0.99) | 0.93 (0.85-1.03) | 1.08 (0.96-1.21) |

Model 1 (basic model): Adjusted for age, sex. Model 4 (fully adjusted model): Adjusted for age, sex, ethnicity, income, education, smoking status, physical.activity, vegetable, fruit, coffee, alcohol intake frequency, BMI, hypertension, diabetes, CVD, and cancer. HR, hazard ratios; BMI, body mass index; CVD, Cardiovascular disease.
