# Supplementary material for: Association of different types of milk with depression and anxiety: a prospective cohort study and Mendelian randomization analysis
Source: Front Nutr. 2024 Dec 5;11:1435435. doi: 10.3389/fnut.2024.1435435 (PMC11656347; doi:10.3389/fnut.2024.1435435)
Supplement: Supplementary file 2 [file Image_1.pdf]

Supplementary Figure 1. Associations of types of milk consumption with anxiety by subgroups

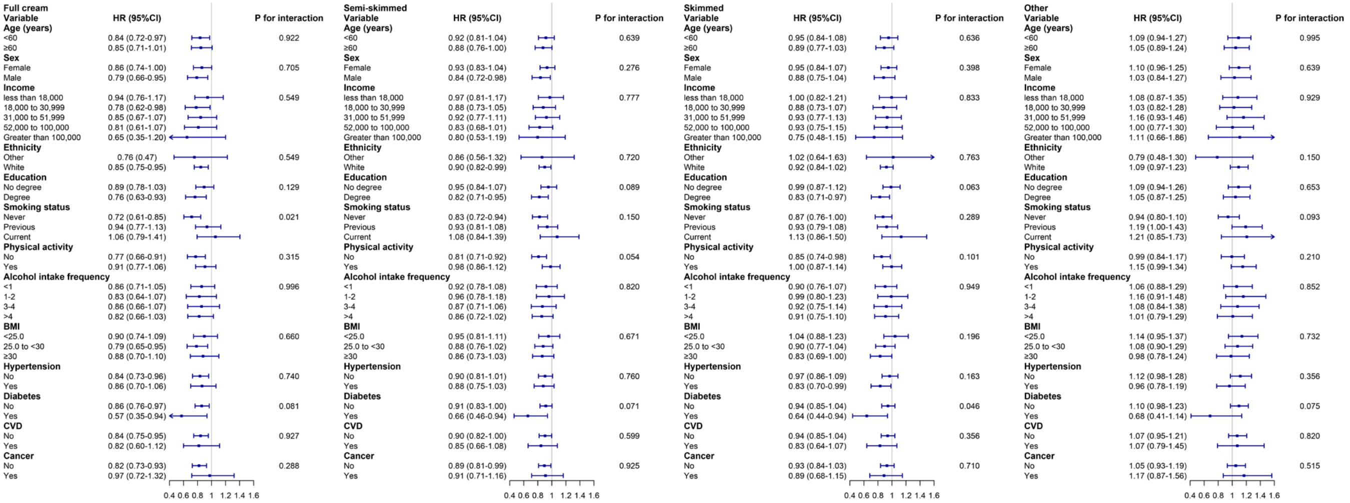

Adjusted for age, sex, ethnicity, income, education, smoking status, physical.activity, vegetable, fruit, coffee, alcohol intake frequency, BMI, hypertension, diabetes, CVD, and cancer. HR, hazard ratios; BMI, body mass index; CVD, Cardiovascular disease.
