# Supplementary material for: Association of different types of milk with depression and anxiety: a prospective cohort study and Mendelian randomization analysis
Source: Front Nutr. 2024 Dec 5;11:1435435. doi: 10.3389/fnut.2024.1435435 (PMC11656347; doi:10.3389/fnut.2024.1435435)
Supplement: Supplementary file 3 [file Image_2.pdf]

Supplementary Figure 2. MR Plots for semi-skimmed milk on anxiety

(A) Scatterplot

(B) Forest plot

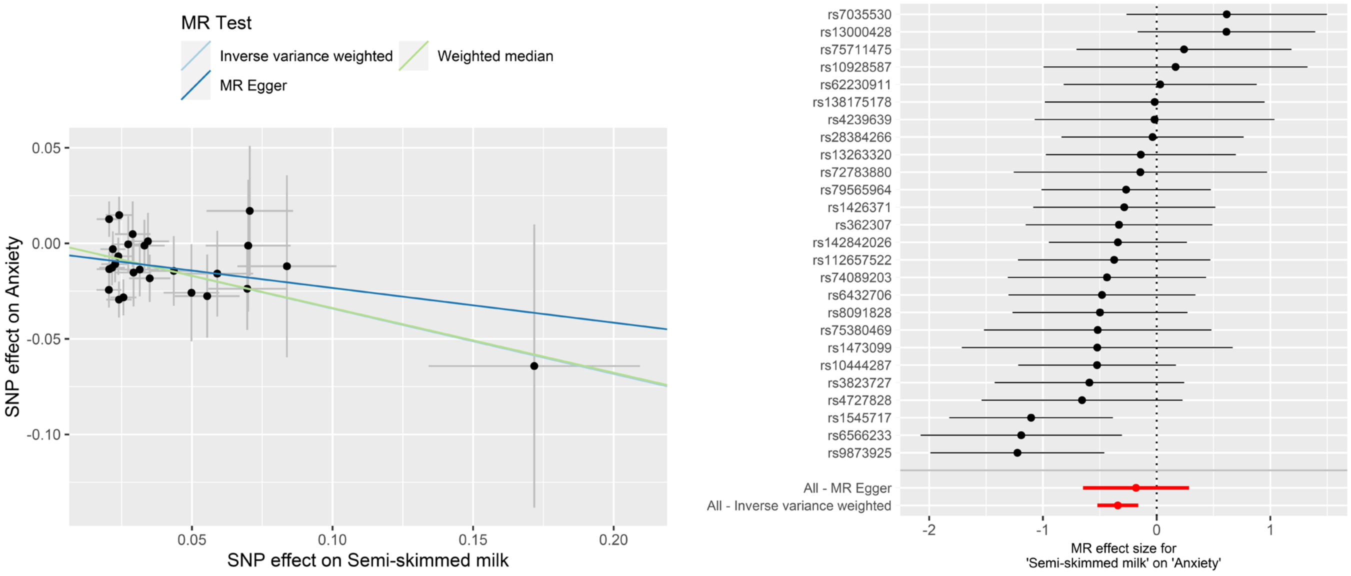

(A) Scatterplot of potential SNP effects on semi-skimmed milk vs anxiety, with the slope of each line corresponding to the estimated MR effect per method. (B) Forest plot of individual and combined SNP MR-estimated effects sizes. MR, mendelian randomization; SNP, single-nucleotide polymorphism.
