# Supplementary material for: Association of different types of milk with depression and anxiety: a prospective cohort study and Mendelian randomization analysis
Source: Front Nutr. 2024 Dec 5;11:1435435. doi: 10.3389/fnut.2024.1435435 (PMC11656347; doi:10.3389/fnut.2024.1435435)
Supplement: Supplementary file 4 [file Image_3.pdf]

Supplementary Figure 3. Leave-one-out MR estimates for the effect of semi-skimmed milk on anxiety

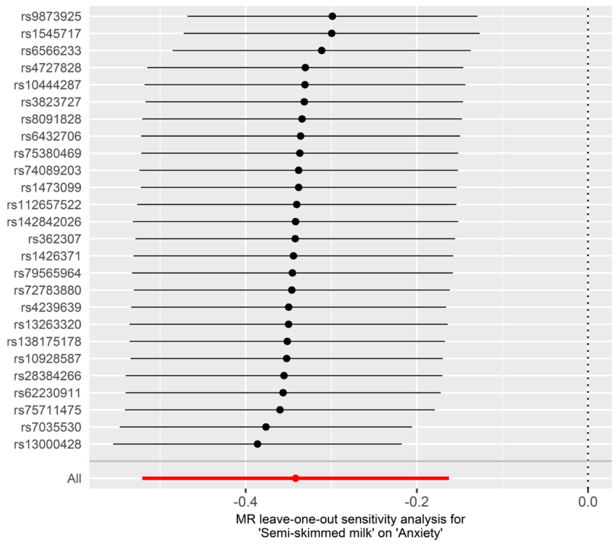

MR, mendelian randomization; SNP, single-nucleotide polymorphism.
