# Supplementary material for: Association of different types of milk with depression and anxiety: a prospective cohort study and Mendelian randomization analysis
Source: Front Nutr. 2024 Dec 5;11:1435435. doi: 10.3389/fnut.2024.1435435 (PMC11656347; doi:10.3389/fnut.2024.1435435)
Supplement: Supplementary file 5 [file Data_Sheet_1.docx]

Association of Different Types of Milk with Depression and Anxiety: A Prospective Cohort Study and Mendelian Randomization Analysis

Chunying Wu, Yusheng Liu, et al.

**Results:** In the UK Biobank cohort, supplementary analyses were conducted by including energy intake as a covariate and excluding participants with missing covariate data. Among the 357,568 participants initially included in the study, 304,814 (85.25%) had missing energy data, leaving 40,543 participants for this analysis. Cox proportional hazards models were applied to assess the relationship between different types of milk consumption and the risk of depression and anxiety.

In the basic model (Model 1, adjusted for age and sex), no significant associations of different milk types with depression were observed. The HR for full cream milk was 1.04 (95% CI: 0.75-1.44), for semi-skimmed milk, it was 0.82 (95% CI: 0.62-1.07), for skimmed milk, it was 0.91 (95% CI: 0.69-1.21), and for other types, it was 1.01 (95% CI: 0.73-1.40). The fully adjusted model (Model 4, adjusted for age, sex, ethnicity, income, education, smoking status, physical activity, vegetables, fruits, coffee, alcohol intake frequency, energy, BMI, hypertension, diabetes, cardiovascular disease, and cancer) also showed no significant associations. The HR for full cream milk was 1.01 (95% CI: 0.72-1.40), for semi-skimmed milk, it was 0.84 (95% CI: 0.64-1.10), for skimmed milk, it was 0.93 (95% CI: 0.70-1.23), and for other types, it was 1.04 (95% CI: 0.75-1.45).

In the basic model (Model 1, adjusted for age and sex), the HR for full cream milk was 0.77 (95% CI: 0.55-1.08), for semi-skimmed milk, it was 0.81 (95% CI: 0.62-1.05), for skimmed milk, it was 0.80 (95% CI: 0.61-1.05), and for other types, it was 0.90 (95% CI: 0.66-1.25), with no significant associations observed between different milk types and anxiety. Similarly, in the fully adjusted model (Model 4, adjusted for age, sex, ethnicity, income, education, smoking status, physical activity, vegetables, fruits, coffee, alcohol intake frequency, energy, BMI, hypertension, diabetes, cardiovascular disease, and cancer), the HR for full cream milk was 0.72 (95% CI: 0.51-1.01), for semi-skimmed milk, it was 0.79 (95% CI: 0.61-1.03), for skimmed milk, it was 0.78 (95% CI: 0.59-1.03), and for other types, it was 0.87 (95% CI: 0.63-1.20). No significant associations were identified.

**Supplementary Analysis**

This file includes:

[Supplementary Table A. The numbers (percentages) of participants with missing covariates...............2](#_Toc31444)

[Supplementary Table B. Association of milk type with risk depression and anxiety in the UK Biobank cohort after adding energy as a covariate and excluding participants with missing covariates 3](#_Toc28412)

# Supplementary Table A. The numbers (percentages) of participants with missing covariates

| **Covariates** | **n** | **%** |
| --- | --- | --- |
| Income | 48,123 | 13.46% |
| Ethnicity | 982 | 0.27% |
| Education | 5,387 | 1.51% |
| Smoking status | 985 | 0.28% |
| Physical activity | 59,807 | 16.73% |
| Vegetable | 1,276 | 0.36% |
| Fruit | 749 | 0.21% |
| Coffee | 344 | 0.10% |
| Alcohol intake frequency | 118 | 0.03% |
| BMI | 1,409 | 0.39% |
| Energy | 304,814 | 85.25% |

BMI, body mass index.

# Supplementary Table B. Association of milk type with risk depression and anxiety in the UK Biobank cohort after adding energy as a covariate and excluding participants with missing covariates

| **Outcomes** | **Non-consumers** | **Milk consumers** | | | |
| --- | --- | --- | --- | --- | --- |
|  |  | **Full cream** | **Semi-skimmed** | **Skimmed** | **Other** |
| **Depression, HR (95% CI)** |  |  |  |  |  |
| Event, *n* (%) | 56 (3.8) | 97 (6.6) | 878 (60.1) | 331 (22.6) | 100 (6.8) |
| Model 1 | 1.00 (Reference) | 1.04 (0.75-1.44) | 0.82 (0.62-1.07) | 0.91 (0.69-1.21) | 1.01 (0.73-1.40) |
| Model 4 | 1.00 (Reference) | 1.01 (0.72-1.40) | 0.84 (0.64-1.10) | 0.93 (0.70-1.23) | 1.04 (0.75-1.45) |
| **Anxiety, HR (95% CI)** |  |  |  |  |  |
| Events, *n* (%) | 60 (4.1) | 76 (5.2) | 926 (62.8) | 316 (21.4) | 97 (6.6) |
| Model 1 | 1.00 (Reference) | 0.77 (0.55-1.08) | 0.81 (0.62-1.05) | 0.80 (0.61-1.05) | 0.90 (0.66-1.25) |
| Model 4 | 1.00 (Reference) | 0.72 (0.51-1.01) | 0.79 (0.61-1.03) | 0.78 (0.59-1.03) | 0.87 (0.63-1.20) |

Model 1 (basic model): Adjusted for age, sex. Model 4 (fully adjusted model): Adjusted for age, sex, ethnicity, income, education, smoking status, physical.activity, vegetable, fruit, coffee, alcohol intake frequency, energy, BMI, hypertension, diabetes, CVD, and cancer. HR, hazard ratios; BMI, body mass index; CVD, Cardiovascular disease.
